# Supplementary material for: Highly efficient serum-free manipulation of miRNA in human NK cells without loss of viability or phenotypic alterations is accomplished with TransIT-TKO
Source: PLoS One. 2020 Apr 17;15(4):e0231664. doi: 10.1371/journal.pone.0231664 (PMC7164639; doi:10.1371/journal.pone.0231664)

**Supplementary Figure 1.** **X-VIVO serum free media best supports NK cell growth for cellular transfections.** NK-92 cells (Top) and RosetteSep isolated primary human NK cells (Bottom) were grown in ATCC recommended media (containing 25% serum) and serum free X-VIVO media for up to 4 days. **A/C)** Images represent magnification with 20x objective lens. **C/D)** Cellular viability were assessed by trypan blue exclusion. Data represents individual (B) or mean (D) values ± standard deviation, n=1-3. Data was assessed by *t* test, ns indicates no significance.


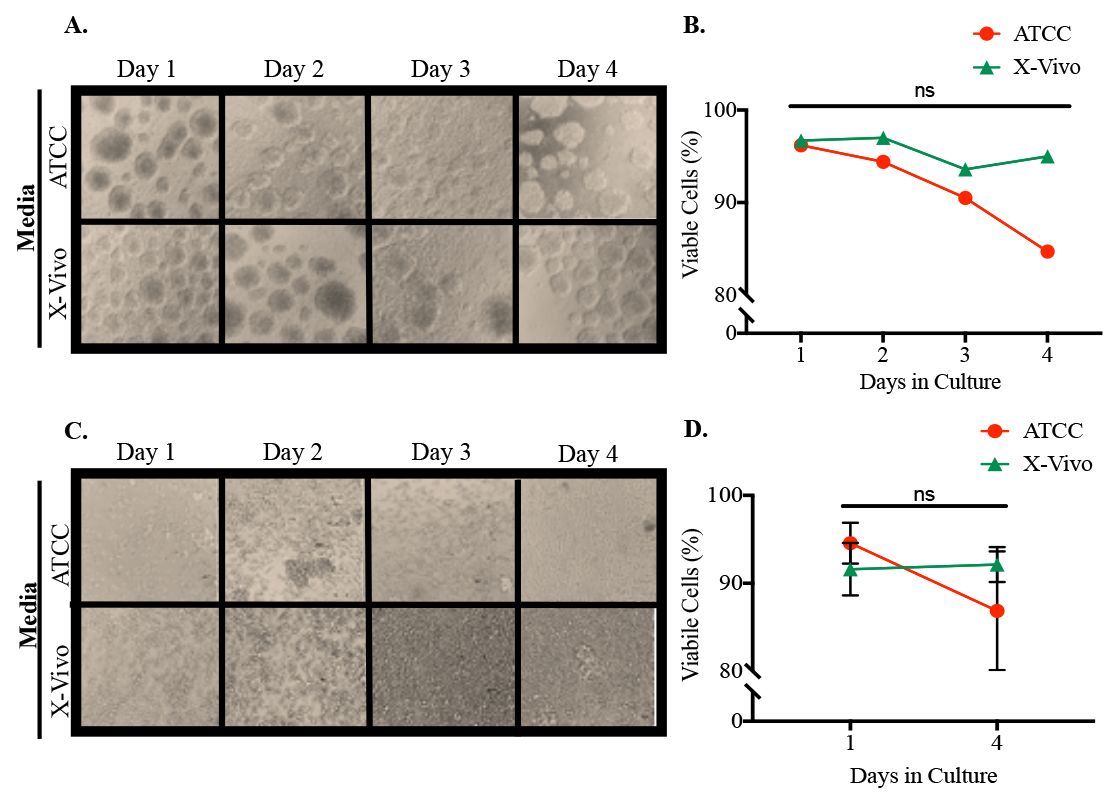

Supplement: S1 Fig — NK-92 cells (Top) and RosetteSep isolated primary human NK cells (Bottom) were grown in ATCC recommended media (containing 25% serum) and serum free X-VIVO media for up to 4 days. A/C) Images represent magnification with 20x objective lens. C/D) Cellular viability were assessed by trypan blue exclusion. Data represents individual (B) or mean (D) values ± standard deviation, n = 1–3. Data was assessed by t test, ns indicates no significance. (DOCX) [file pone.0231664.s001.docx]
