# Supplementary material for: Highly efficient serum-free manipulation of miRNA in human NK cells without loss of viability or phenotypic alterations is accomplished with TransIT-TKO
Source: PLoS One. 2020 Apr 17;15(4):e0231664. doi: 10.1371/journal.pone.0231664 (PMC7164639; doi:10.1371/journal.pone.0231664)
Supplement: S2 Fig — RosetteSep isolated primary human NK cells (A) primary human PBMCs (B) and primary JIA FLS (C) were transfected with miR-146a-5p sense or antisense miRNA compared to non-transfected control cells. A-C) Cellular viability, purity, and efficiency were determined by flow cytometry. D) MiRNA delivery was assessed by RTqPCR. Baseline reflects expression level of mi-146a-5p in cells transfected with negative control miRNA. Fold changes compared to negative were calculated using two reference miRNAs and the Pflaff Method. Data represents individual measurements and bars represent mean ± standard deviation, n = 1–3. RTqPCR results were assessed by one-way ratio paired t tests. (DOCX) [file pone.0231664.s002.docx]

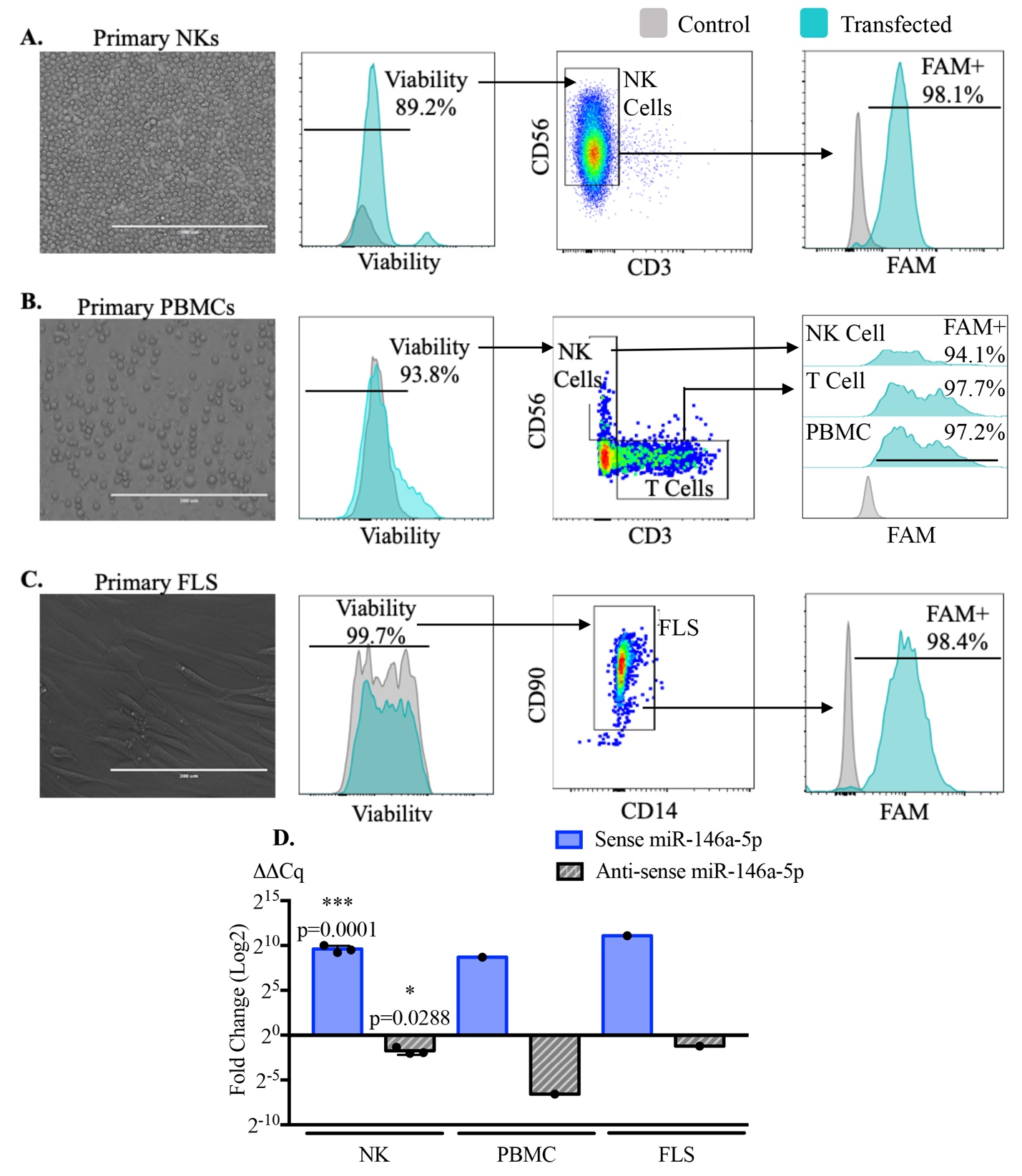


**Supplementary Figure 2. TransIT-TKO is effective for transfection of lymphocytes and fibroblasts.** RosetteSep isolated primary human NK cells (**A**) primary human PBMCs (**B**) and primary JIA FLS (**C**) were transfected with miR-146a-5p sense or antisense miRNA compared to non-transfected control cells. **A-C)** Cellular viability, purity, and efficiency were determined by flow cytometry. **D)** MiRNA delivery was assessed by RTqPCR. Baseline reflects expression level of mi-146a-5p in cells transfected with negative control miRNA. Fold changes compared to negative were calculated using two reference miRNAs and the Pflaff Method. Data represents individual measurements and bars represent mean ± standard deviation, n=1-3. RTqPCR results were assessed by one-way ratio paired *t* tests.
