# Supplementary figures and images for: Highly efficient serum-free manipulation of miRNA in human NK cells without loss of viability or phenotypic alterations is accomplished with TransIT-TKO
Source: PLoS One. 2020 Apr 17;15(4):e0231664. doi: 10.1371/journal.pone.0231664 (PMC7164639; doi:10.1371/journal.pone.0231664)

**
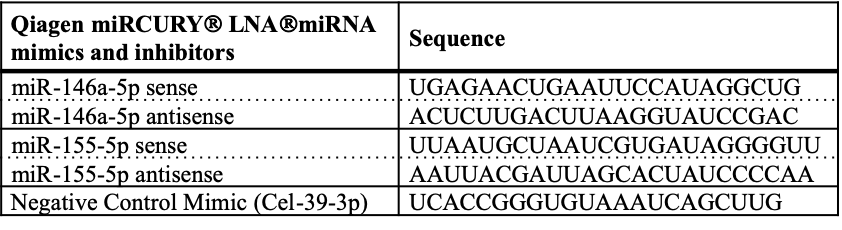
**

**Supplementary Table 1. Qiagen miRCURY LNA sense and antisense miRNA sequences.**

Supplement: S1 Table — (DOCX) [file pone.0231664.s003.docx]

**Supplementary Table 2. Primer sequences, efficiencies, and annealing temperatures for miRNA.**

**
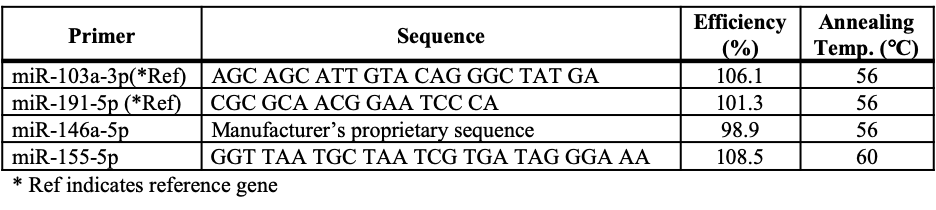
**

Supplement: S2 Table — (DOCX) [file pone.0231664.s004.docx]
