# Supplementary material for: Highly efficient serum-free manipulation of miRNA in human NK cells without loss of viability or phenotypic alterations is accomplished with TransIT-TKO
Source: PLoS One. 2020 Apr 17;15(4):e0231664. doi: 10.1371/journal.pone.0231664 (PMC7164639; doi:10.1371/journal.pone.0231664)
Supplement: S3 Table — (DOCX) [file pone.0231664.s005.docx]

**Supplementary Table 3. Primer sequences, efficiencies, and annealing temperatures for mRNA.**

| **Primer** | **Sequence** | **Efficiency (%)** | **Annealing**  **Temp (°C)** |
| --- | --- | --- | --- |
| **HPRT1 (ref)** | Manufacturer's Proprietary Sequence - Bio-Rad | 109.3% | 60^o^C |
| **GAPDH (ref)** | Manufacturer's Proprietary Sequence - Bio-Rad | 99.6% | 60^o^C |
| **IRAK1** | Manufacturer's Proprietary Sequence - Bio-Rad | 99.1% | 60^o^C |
| **STAT1** | Forward: ATG GCA GTC TGG CGG CTG AAT T | 102.1% | 60^o^C |
|  | Reverse: CCA AAC CAG GCT GGC ACA ATT G |  |  |
