# Supplementary material for: Highly efficient serum-free manipulation of miRNA in human NK cells without loss of viability or phenotypic alterations is accomplished with TransIT-TKO
Source: PLoS One. 2020 Apr 17;15(4):e0231664. doi: 10.1371/journal.pone.0231664 (PMC7164639; doi:10.1371/journal.pone.0231664)
Supplement: S4 Table — (DOCX) [file pone.0231664.s006.docx]

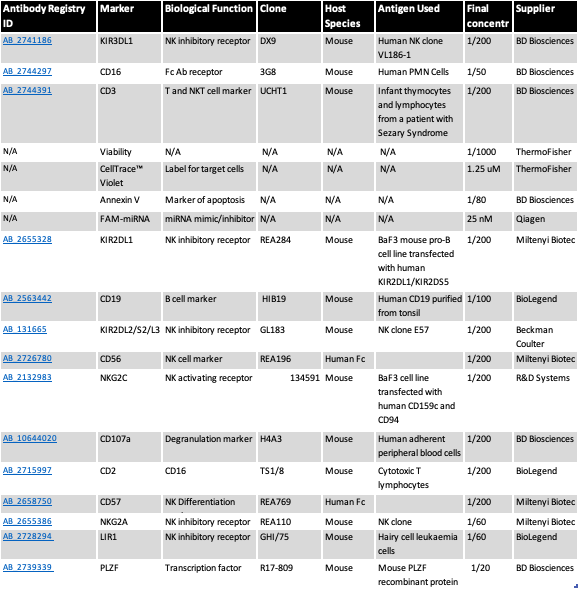


**Supplementary Table 4. Flow Cytometry Antibodies, dyes and labels.**

All antibodies were monoclonal.
