# Supplementary material for: Highly efficient serum-free manipulation of miRNA in human NK cells without loss of viability or phenotypic alterations is accomplished with TransIT-TKO
Source: PLoS One. 2020 Apr 17;15(4):e0231664. doi: 10.1371/journal.pone.0231664 (PMC7164639; doi:10.1371/journal.pone.0231664)
Supplement: S5 Table — (DOCX) [file pone.0231664.s007.docx]

**Supplementary Table 5. Non-exhaustive MiRBase sequence blast.**
